# Association Between Single-Nucleotide Polymorphisms in *Toll-like Receptor 3* (*tlr3*), *tlr7*, *tlr8* and *tirap* Genes with Severe Symptoms in Children Presenting COVID-19

**DOI:** 10.3390/v17010035

**Published:** 2024-12-30

**Authors:** Adriana Souza Andrade, Aline Almeida Bentes, Lilian Martins Diniz, Silvia Hees Carvalho, Erna Geessien Kroon, Marco Antonio Campos

**Affiliations:** 1 Instituto René Rachou/Fiocruz Minas, Belo Horizonte 30190-009, MG, Brazil; andradeadriana.ds@gmail.com (A.S.A.); silviahees@outlook.com (S.H.C.); 2 Departamento de Pediatria, Universidade Federal de Minas Gerais, Belo Horizonte 30130-110, MG, Brazil; alinebentes2000@gmail.com (A.A.B.); lilianmodiniz@gmail.com (L.M.D.); 3 Hospital Infantil João Paulo II, Minas Gerais, Belo Horizonte 30130-110, MG, Brazil; 4 Laboratório de Vírus, Departamento de Microbiologia, Universidade Federal de Minas Gerais, Minas Gerais, Belo Horizonte 31270-201, MG, Brazil; ernagkroon@gmail.com

**Keywords:** single-nucleotide polymorphisms, genes innate immunity, SARS-CoV2, symptom severity in children presenting COVID-19

## Abstract

The global number of COVID-19 deaths has reached 7 million, with 4% of these deaths occurring in children and adolescents. In Brazil, around 1500 children up to 11 years old died from the disease. The most common symptoms in children are respiratory, potentially progressing to severe illnesses, such as severe acute respiratory syndrome (SARS) and MIS-C. Studies indicate that comorbidities and genetic factors, such as polymorphisms in immune response genes, can influence the severity of COVID-19. This study investigates the occurrence of single-nucleotide polymorphisms (SNPs) in innate immune response genes in children with COVID-19. Seventy-three samples were analyzed from children under 13 years old hospitalized at João Paulo II Children’s Hospital due to COVID-19. The evaluated SNPs were *tlr8 (1)* (rs3764879), *tlr8 (2)* (rs2407992), *tlr7* (rs179008), *tlr3* (rs3775291), *tirap* (rs8177374), and *mcp-1* (rs1024611), considering four categories of severity: mild, moderate, severe, and critical COVID-19. To identify the SNPs, PCR and sequencing were performed. The frequencies of the SNPs obtained were not discrepant when compared to the frequencies described in the Global ALFA, Global 1000 Genomes, Global gnomAD, American 1000 Genomes, and American gnomAD databases, except for the SNP in TLR7. Comparing severe and critical cases to mild and moderate cases, we found a higher relative risk associated with mutations in *tlr8 (1)*, *tlr7*, *tlr3*, and *tirap* (*p* < 0.05). No association was found for SNPs in *tlr8 (2)* and *mcp-1*. Our analyses suggest an association between SNPs in innate immune response genes and severity of symptoms in children with COVID-19 (or SARS-CoV-2 infected children).

## 1. Introduction

From the start of the pandemic to mid-2024, the global number of COVID-19 cases surpassed 800 million, resulting in over 7 million deaths. Among these, approximately 4% (around 280,000) were children and adolescents under the age of 20 [1,2]. In Brazil, 38,984,103 COVID-19 cases and 713,966 COVID-19 deaths have been confirmed [3]. Among these fatalities, approximately 1500 involved children up to 11 years old. Additionally, there were over 2400 reported cases of multisystem inflammatory syndrome in children (MIS-C) associated with COVID-19. By 2022, COVID-19 ranked among Brazil’s top 10 leading causes of child mortality [4]. The most common acute symptoms in children with COVID-19 affect the respiratory tract, including fever, cough, headache, and myalgia [5]. However, as the disease progresses, a variety of other symptoms may emerge, such as severe acute respiratory syndrome (SARS), necrotizing pneumonia, cardiovascular and neurological manifestations, and MIS-C, all of which can lead to death [6].

Since the onset of the pandemic, an association has been observed between severe COVID-19 cases and the presence of comorbidities in adults [7]. However, severe cases have also been reported in children without any pre-existing medical conditions. The literature indicates that children have developed severe forms of the disease even in the absence of comorbidities, leading to death or, in other cases, irreversible sequelae [8,9,10]. The factors influencing a favorable prognosis versus a fatal outcome in COVID-19 cases include the individual’s immune response, among other components. Research, including findings by Zhang et al. (2022) [11], indicates that genetic conditions affecting immune mediators, like type I interferon, can significantly influence disease progression. These conditions may be responsible for about 10% of COVID-19 hospitalizations in children.

Understanding this pathogen–host dynamic also requires knowledge of the viral component. The etiological agent of COVID-19, the *Betacoronavirus pandemicum* (SARS-CoV-2), has a positive-sense single-stranded RNA genome of approximately 30 kb. The virus replicates in the cell’s cytoplasm, where its genetic material and proteins are synthesized in different regions of the cytoplasm [12]. The cellular tropism of SARS-CoV-2 is primarily determined by the co-expression of ACE2 and TMPRSS2 receptors, which are found in cells from various tissue types, including ciliated and secretory nasal cells, type II alveolar epithelial cells, enterocytes in the small and large intestines, proximal tubular cells of the kidney, cardiomyocytes, and vascular endothelial cells [6]. Campos et al. (2021) [13] suggest that testicular damage observed in severe COVID-19 cases may be partially explained by direct infection of testicular cells by SARS-CoV-2.

During the initial contact with an individual’s organism, viral components, and other pathogen-associated molecular patterns (PAMPs) are recognized by the early components of the immune response, the innate immune response, through pattern recognition receptors (PRRs), including toll-like receptors (TLRs) [8,14,15,16,17,18]. The following mutations were evaluated: *tlr8* (rs3764879 and rs2407992), *tlr7* (rs179008), *tlr3* (rs3775291), *tirap* (rs8177374), and *mcp-1* (rs1024611). TLR3, TLR7, and TLR8 receptors are located on the membranes of endosomes. When activated, TLRs trigger a phosphorylation cascade, culminating in the nuclear translocation of NFκB, which activates the expression of pro-inflammatory genes, including interleukins, cytokines, and type I interferons (IFN I) and interferon regulatory factors (IRFs), which further stimulate the expression of IFN I. TLRs also activate immune cells, such as dendritic cells and macrophages, and orchestrate the subsequent induction of the adaptive immune response [14,15,16,19]. TLR7 and TLR8 recognize single-stranded RNA (ssRNA), while TLR3 recognizes double-stranded RNA (dsRNA) [14,15]. These ligands are often derived from viruses, highlighting the role of these receptors in antiviral defense, particularly against RNA viruses such as SARS-CoV-2 [8,9,11,20,21].

MCP-1 (monocyte chemoattractant protein-1), also known as CCL2, is a chemokine that directs the migration and infiltration of monocytes, macrophages, and microglia to the injury site. It plays a crucial role in regulating the intensity of inflammation and activating these cells’ ability to phagocytize pathogens and produce cytokines [8,19,22]. TIRAP (toll-interleukin 1 receptor (TIR) domain-containing adapter protein) is an adaptor protein involved in signaling specific TLRs. TIRAP is an intermediary that links TLR activation to the signaling cascade, leading to cytokine production and immune cell activation. Both MCP-1 and TIRAP play critical roles in activating and regulating the innate immune response [8,22,23,24,25].

Several isoforms of *tlrs* result from single-nucleotide base mutations in the coding genes, known as single-nucleotide polymorphisms (SNPs). Some of these alterations can lead to loss-of-function (LOF), hypofunctional, or hypomorphic polymorphisms, which can impair the inflammatory response and increase an individual’s susceptibility to certain diseases, including COVID-19 [8,9,11,26,27]. Bagheri-Hosseinabadi et al. (2022) [28] showed that TLR3, TLR7, TLR8, and TLR9 were associated with the response to COVID-19 and that hyperexpression of these receptors in respiratory epithelial cells from COVID-19 patients was linked to increased disease severity. Therefore, since these components of the innate immune response are related to the response to COVID-19, what would be the impact of a deficiency in their functions? Zhang et al. (2022) [11] report that genetic errors leading to deficiencies in type I IFN might account for approximately 10% of hospitalizations for COVID-19 pneumonia in children. SNPs represent the most common genetic variation among individuals within a species. These variations can be linked to enzymatic deficiencies or defects in the function associated with the innate immune response [11]. Such genetic errors may indicate an increased risk of developing severe COVID-19 and could serve as potential markers for both poor and favorable prognoses.

All the SNPs selected for the study influence gene function and expression or alter protein functionality. However, only some studies have sought to understand the underlying causes of these impacts. The SNP rs3764879, 129 G>C, located in the *tlr8* gene promoter region, is associated with changes in *tlr8* transcriptional activity, thereby influencing its expression [29]. The SNP rs2407992 G>C is found in a coding region, and its effects have been evaluated through in silico analyses. Although this is a synonymous SNP, it may affect splicing by abolishing a splicing activator domain, potentially leading to changes in the expression of the TLR8 protein and, consequently, variations in the immune response [29,30]. The SNP rs179008 A>T in exon 3 of the *tlr7* gene results in the substitution of glutamine (Gln) to leucine (Leu) at position 11 of the TLR7 protein, causing a loss of function [11]. The SNP rs2407992 G>C is found in a coding region, and its effects have been evaluated through in silico analyses [11]. Although this is a synonymous SNP, it may affect splicing by abolishing a splicing activator domain, potentially leading to changes in the expression of the TLR8 protein and, consequently, variations in the immune response [29,30]. The SNP rs3775291 C>T in *tlr3* is a non-synonymous mutation at position 412, changing leucine (Leu) to phenylalanine (Phe). The SNP rs8177374 C>T results in the substitution of serine (Ser) to leucine (Leu) at position 180 in the tirap gene. Finally, the SNP rs1024611 A>G, located in the distal regulatory region of the *mcp1* gene at position −2518 relative to the transcription start site, could influence transcriptional activity due to an allelic expression imbalance and the preferential transcription of the G allele [29,30].

Therefore, studies are necessary to explore the potential relationships between severe COVID-19 and the presence of the host’s SNPs related to innate immunity. The primary aim of this study is to investigate the occurrence of SNPs in genes essential for the innate immune response in children infected with SARS-CoV-2 and to assess the potential association of these SNPs with more severe manifestations of COVID-19.

## 2. Material and Methods

### 2.1. Declarations

The Institute René Rachou Ethical Committee, Fiocruz, CAAE 37207920.6.0000.5091 and Fundação Hospitalar do Estado de Minas Gerais—FHEMIG, CAAE 37207920.6.3001.5119 approved this project. The patients’ responsible written permission was obtained.

### 2.2. Study

#### 2.2.1. Design and Sampling

This cross-sectional study was conducted with data collected between 2021 and 2022, focusing on children under 13 years old hospitalized at the Hospital Infantil João Paulo II (Pediatric Hospital) during the COVID-19 epidemic. The hospital team collected information on the children’s clinical conditions and total blood samples following the inclusion and exclusion criteria defined in the project. Inclusion criteria included children hospitalized with symptoms of infection, diagnosed with COVID-19 via laboratory tests (including PCR, antigen tests, and IgM/IgG antibody detection), whose parents consented to participate in the research by signing the informed consent form (ICF), and, when necessary, the informed assent form (IAF). Exclusion criteria included children previously on immunosuppressive medication or those who have had infections in a state of immunosuppression.

After the screening process, which involved the analysis of clinical data, elimination of duplicate samples, and those without laboratory confirmation of infection, a total of 73 samples were selected for the study. Total blood samples were fractionated by centrifugation into cellular concentrate and serum and stored at −80 °C in the biobank of the Laboratório de Imunologia de Doenças Virais, Instituto René Rachou, Fundação Oswaldo Cruz. The cellular concentrate was used to identify possible mutations in children’s components of the innate immune response using SANGER sequencing techniques [31]. The analysis steps up to sequencing included chromosomal DNA extraction, polymerase chain reaction (PCR), electrophoresis, and purification of the amplified material.

#### 2.2.2. DNA Extraction, Amplification, and Sequencing

Genomic DNA extraction was performed using the DNeasy Blood and Tissue Kit (Qiagen, Germany). PCRs for the amplification of DNA fragments were conducted for mutations located in the *tlr8* [rs3764879 (1) and rs2407992 (2)], *tlr7* (rs179008), *tlr3* (rs3775291), *tirap* (rs8177374), and *mcp-1* (rs1024611) genes, using the primers described in Table 1. Primers were designed using the Oligo Analyzer algorithm provided by Integrated DNA Technologies (IDT) at the following web address: https://www.idtdna.com/calc/analyzer, accessed on 2 February 2021. Pairing temperatures were selected between 50° and 58 °C, with an amplified fragment size of up to 500 base pairs. For each reaction, approximately 100 ng of DNA and 0.25 µL of each primer at 10 mM were used, along with 10 µL of GoTaq^®^ Probe qPCR Master Mix (Promega, Madison, WI, USA) and water to a final volume of 20 µL. Amplifications were performed using a thermocycler (Eppendorf, Hamburg, Germany) with the following temperature variations: initial denaturation at 94 °C for 2 min, 40 cycles of denaturation at 94 °C for 30 s, annealing at 50–58 °C (depending on the gene) for 30 s, and extension at 72 °C for 30 s. This process was concluded with a final extension at 72 °C for 1 min. To analyze the amplification of DNA fragments, 2 µL of the reaction was loaded onto a 2% agarose (Medchemexpress, Monmouth Junction, NJ, USA). Gel Electrophoresis was conducted using 1X TAE buffer (Tris 40 mM (Advancion Sciences, Buffalo Grove, LA, USA), acetic acid 20 mM (Celanese Corporation, Irving, TX, USA), and EDTA 1 mM (Dow Chemical Company, Midland, MI, USA)) at 110 V. The gel was stained with SYBR Safe (Thermo Fisher Scientific, Waltham, MA, USA) for 30 min and visualized under UV light. Amplified DNA purification was performed using PCR products and either the Gel Band Purification Kit (GE Healthcare, Chicago, IL, USA) or QIAquick PCR Purification Kit (Qiagen, Hilden, Germany), following the manufacturer’s specifications. Forward and reverse primers were added separately to the DNA fragments in 96-well plates. Sequencing was performed by the René Rachou Institute sequencing department using the Sanger method [31] with an ABI 3730xL 48-capillary sequencer.

#### 2.2.3. Sequencing Analysis

Sequencing data were analyzed using the SeqTrace [35]. Forward and reverse sequences of each sample were aligned complementarily and examined for mutation sites. The location of each analyzed site and the expected mutation are described in Table 1. The quality of the amplified region was assessed, and sequencing was repeated if necessary. For homozygous mutations, nucleotide readings were conducted as usual. In the case of heterozygous mutations, a sample was considered positive for the mutation if two nucleotide signals overlap at the mutation site. X-linked mutations appear as a single allele in male children, with no heterozygosity observed in these cases. After identifying the mutations, the data were plotted in the data collection tables. Statistical data analysis was performed with 73 samples.

#### 2.2.4. Criteria for Severity Classification

The severity levels were classified based on children’s clinical characteristics, using specific criteria for different conditions.

Children with respiratory symptoms: Classification for these children followed the guidelines established by ’The PODIUM Consensus Conference’ [36] and was divided into four categories, as follows. Mild included children who did not require oxygen supplementation and had no associated comorbidities. Moderate included children who required oxygen via nasal catheter, high flow, or mask with flow up to 6 L or who had comorbidities. Severe included children who needed an oxygen with a mask at flows greater than 6 L, non-invasive ventilation (NIV), CPAP (continuous positive airway pressure), BIPAP (bilevel positive airway pressure), or required admission to an Intensive Care Unit (ICU). Critical included cases where ICU admission with mechanical ventilation support was necessary.

Patients with Pediatric Multisystem Inflammatory Syndrome (MIS-C) and Kawasaki: For these patients, severity was classified into three levels. Moderate: patients who did not require ICU admission. Severe: patients who required ICU admission. Critical: cases requiring vasopressors, inotropes, or mechanical ventilation support.

Patients with other clinical manifestations (such as hepatitis or neurological conditions) and who used programs: classification for these manifestations followed these criteria. Severe included patients who did not require ICU admission. Critical involved cases that required ICU admission or resulted in death.

These criteria enabled a thorough assessment of case severity, enhancing comparative analysis and our understanding of the various clinical profiles of the children involved in the study.

#### 2.2.5. Statistical Analysis

We used Microsoft Excel 365 to create pie charts, bar charts, or Venn diagrams. Statistical correlation analysis was performed using Fisher’s exact test [37], with a significance level set at *p* < 0.05. Relative risk calculations were conducted using Koopman’s asymptotic score [38]. Box plots, violin plots, heat maps, and forest plots were generated using GraphPad Prism 8., GraphPad Software, Inc., San Diego, CA, USA.

## 3. Results

### 3.1. Characterization of Hospitalized Children with COVID-19: Age-Related Vulnerabilities, Comorbidities, and Diagnostic Methods

**Figure 1:**
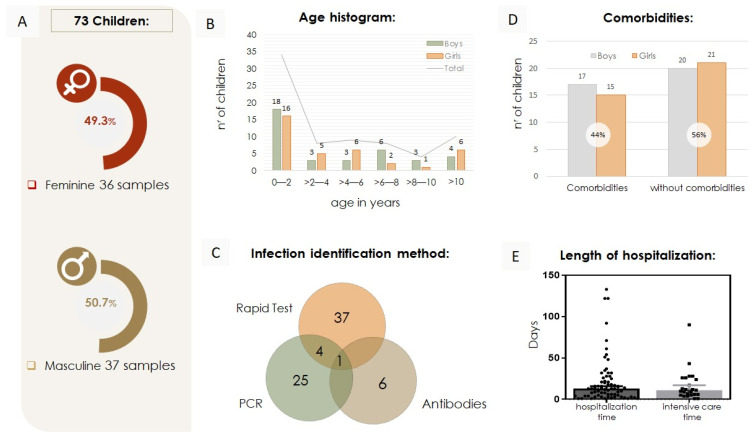
Percentage and age of infected boys and girls with or without comorbidities, hospitalization duration, and diagnosis methods. (**A**) Percentage of children by sex out of 73 samples. (**B**) Age histogram of infected children, showing boys and girls separately, from 0–2, > 2–4, >4–6, >6–8, > 8–10, and >10 years old. The most vulnerable age group was under 2 years old. (**C**) Methods employed to diagnose SARS-CoV-2 infection in the samples studied. (**D**) A total of 73 infected children, boys, and girls, with or without comorbidities. (**E**) The median length of hospitalization and intensive care stay, was 21 and 16 days, respectively.

Among the 73 samples studied, 36 (49.31%) were girls, and 37 (50.7%) were boys (Figure 1A). The children’s ages ranged from just under 13 years (see Figure 1B). As illustrated in Figure 1B, the most affected age group was children under 2 years old, accounting for 46,6% (34 samples). However, the symptoms of COVID-19 were observed in all age groups. COVID-19 diagnoses in these children were confirmed using various laboratory techniques, including PCR, antigen tests, and IgM/IgG antibody detection. Most children, 57.5% (42 samples), were primarily diagnosed using rapid tests alone or in combination with other methods. The remaining samples were diagnosed through PCR and antibody detection, as shown in Figure 1C. Comorbidities were identified in 32 children (43.8%), as shown in Figure 1D. These comorbidities included various conditions, including asthma, sickle cell anemia [9], heart disease, and trisomy 21. The length of hospital stay ranged from 1 day to approximately 4.5 months, with a median hospitalization time of 13 days. Regarding intensive care, 18 children (24.6%) required admission to the Intensive Care Unit (ICU), with stays varying from 1 day to around 3 months and a median duration of 11 days, as depicted in Figure 1E.

### 3.2. Symptom Overlap and Clinical Outcomes in Pediatric COVID-19: Symptomatology, Severity, and Mortality Analysis

**Figure 2:**
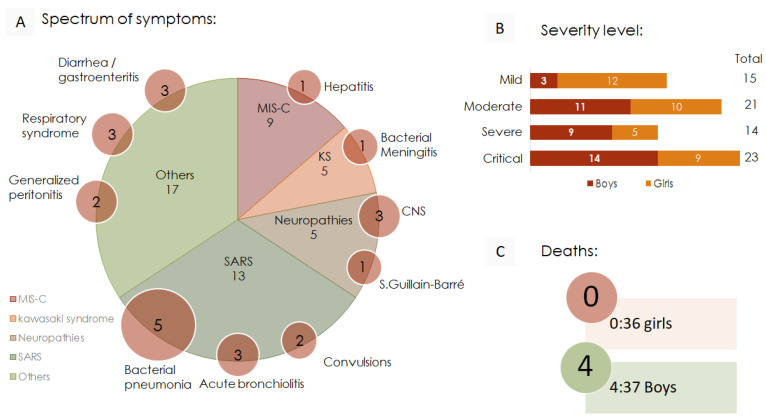
Clinical evolution and severity analysis in pediatric COVID-19. (**A**) Spectrum of symptoms in 73 children. The symptoms presented with multiple simultaneous manifestations. The red circles indicate the number of symptoms overlapping with the primary diagnosis. Primary diagnoses included MIS-C (multisystem inflammatory syndrome in children), Kawasaki syndrome (KS), neuropathies in SARS-CoV-2-positive infections (CNS = central nervous system), SARS (severe acute respiratory syndrome), and others, each presenting a wide variety of clinical symptoms. (**B**) Severity level according to the PODIUM [37] hospital classification. (**C**) Number of deaths and their frequencies within each sex (number of deaths–number of samples).

The symptoms presented by the 73 children, described in Figure 2A, were highly diverse and complex, affecting multiple organs and systems. The most common symptoms included SARS in 13 children (31.5%), MIS-C in 9 children (13.7%), neuropathies associated with SARS-CoV-2 infections in 5 children (12.3%), Kawasaki syndrome (KS) in 5 children (8.2%), and varied cases included in “Others” in 17 children (34.2%). However, these clinical presentations often occurred alongside other simultaneous symptoms, such as opportunistic bacterial infections, hepatitis, and various neurological symptoms, including Guillain–Barré syndrome, ataxia, seizures, and meningitis. In addition to respiratory syndrome, gastroenteritis, and peritonitis, “others” are included in cases such as acute generalized lymphadenitis, acute renal failure, and otitis media. Using the severity analysis based on the Pediatric Organ Dysfunction Information Update Mandate (PODIUM) consensus [36] and the hospital’s standardization for case description, the cases were categorized into four levels of criticality; 15 children were classified as mild, 21 as moderate, 14 as severe, and 23 as critical. Notably, the deaths were included in the critical category. The proportion of boys and girls in each category is shown in Figure 2B. In total, there were 4 deaths, resulting in a mortality frequency of approximately 1 out of every 18 hospitalized children. Another significant observation is that all the deaths occurred exclusively among boys (Figure 2C).

### 3.3. Comparison of SNP Allelic Frequencies in Children with COVID-19: Population and Sex-Based Variations

**Figure 3:**
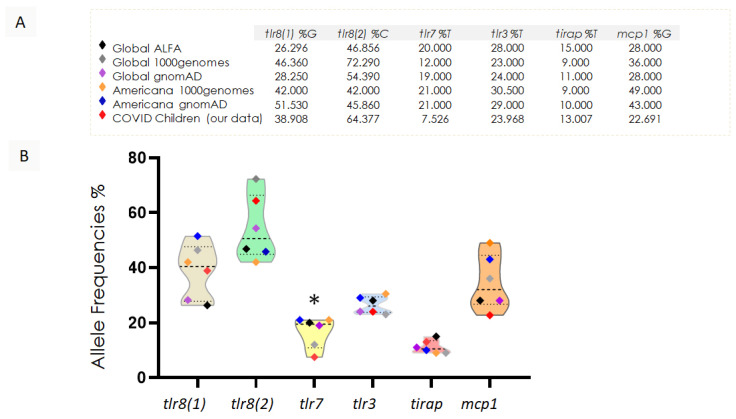
Comparative analysis of SNPs allelic frequencies in pediatric COVID-19 hospitalizations. (**A**) Five public database references of random populations and our work group’s percentage of SNPs from *tlr8 (1)*, *tlr8 (2)*, *tlr7*, *tlr3*, *tirap*, and *mcp1*. (**B**) Allelic frequency of the polymorphisms found in the 73 samples from hospitalized children with COVID-19 compared to (**A**). In dashed lines, the violin plot shows the media and the first and third quartiles. * Statistically significant frequency differences were observed from the four database groups (except for Global 1000 genomes) to *tlr7*.

The allelic frequencies of SNPs in the coding genes *tlr8 (1)*, *tlr8 (2)*, *tlr7*, *tlr3*, *tirap*, and *mcp1* were calculated for the population of hospitalized children with COVID-19 and compared with global databases such as Global ALFA, Global 1000 genomes, and Global gnomAD, as well as databases with populations with genotypes closer to South America, such as Americana 1000 Genomes and Americana genomAD (dbSNP (https://www.ncbi.nlm.nih.gov/snp/, accessed on 15 July 2024). In some genes, such as *tlr7*, *tlr3*, and *mcp1*, the frequencies of SNPs in hospitalized children with COVID-19 were lower than those described in other databases, as shown in Figure 3. There was no statistical difference in the finding of SNPs in trl7 between the Global 1000 genomes and our study group.

**Figure 4:**
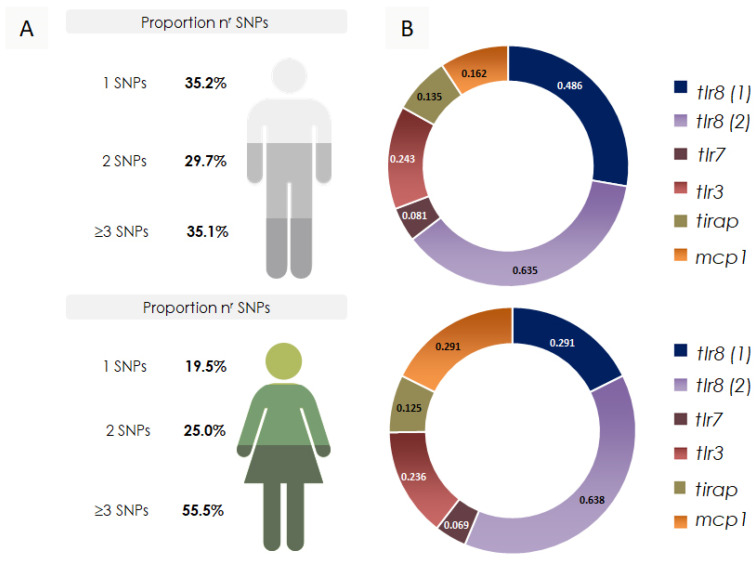
Concentration of single-nucleotide polymorphisms (SNPs) in the study population. (**A**) This figure illustrates the overall concentration of the six SNPs studied, emphasizing their density based on sex. (**B**) The distribution of each SNPs according to sex is shown in the graphs, which highlight any potential differences in SNP frequency and presence between males and females.

The analysis of the concentration of polymorphisms in genes encoding the receptors TLR8 (SNPs 1 and 2), TLR, and TLR3 and the mediators MCP1 and TIRAP revealed that girls have a higher cumulative number of these polymorphisms. It is important to say that the genes *tlr7* and *tlr8* are in chromosome X. Since girls have two X chromosomes while boys have only one, girls have the potential to be either homozygous or heterozygous for these polymorphisms (Figure 4). The distribution of *tlr8 (1)* in girls is lower than the distribution in boys, whereas the distribution of *mcp1* is higher in girls than in boys.

### 3.4. Analysis of SNP Frequency Distribution Based on Severity of Symptoms in Children Presenting COVID-19

**Figure 5:**
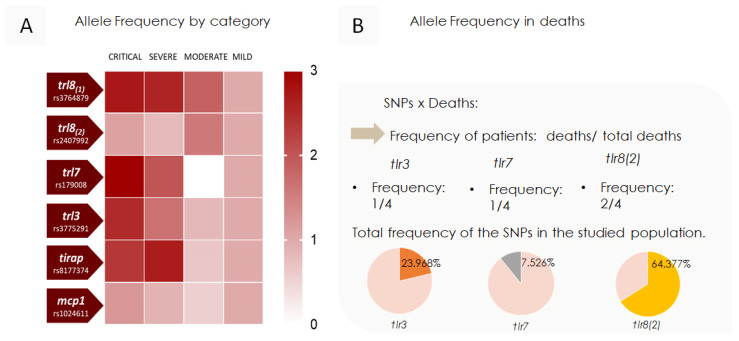
The ratio of allele frequency distribution of the polymorphisms compared to mild symptom cases. (**A**) The allelic frequency distribution of the polymorphisms was categorized based on symptom severity according to the Pediatric Organ Dysfunction Information Update Mandate (PODIUM). Color gradations were used, with lighter shades representing lower frequencies and darker shades indicating higher frequencies. (**B**) The frequency distribution of SNPs found in four fatalities was compared to the overall frequency found in the population studied.

The distribution of SNPs’ frequencies was analyzed according to the symptom severity category, as depicted in Figure 5. It was observed that there was an increase in color intensity in more severe symptom cases for SNPs *tlr8 (1)*, *tlr7*, *tlr3*, and *tirap*. The SNP *tlr8 (1)* showed a proportional gradient relative to symptom severity. In contrast, *tlr7*, *tlr3*, and *tirap* had an SNP frequency distribution primarily concentrated in the severe and critical symptom categories. Notably, *tlr7* was almost exclusively present in the severe and critical symptom categories, with only one case identified in the mild symptom category, which involved a heterozygous girl. For the SNPs *tlr8 (2)* and *mcp1*, frequencies appeared randomly distributed across the categories, with varying intensities observed among different symptom severity levels. Regarding fatal cases, one of four individuals had the mutation for *tlr7*, one for *tlr3*, and two for *tlr8 (2)*. These data, along with the total frequency distribution of these SNPs found in fatalities, have been included in Figure 5B to assess the probability of these occurrences being random or statistically significant.

### 3.5. Impact of SNP Mutations on Severity of Symptoms in Children Presenting COVID-19: Risk Analysis

**Figure 6:**
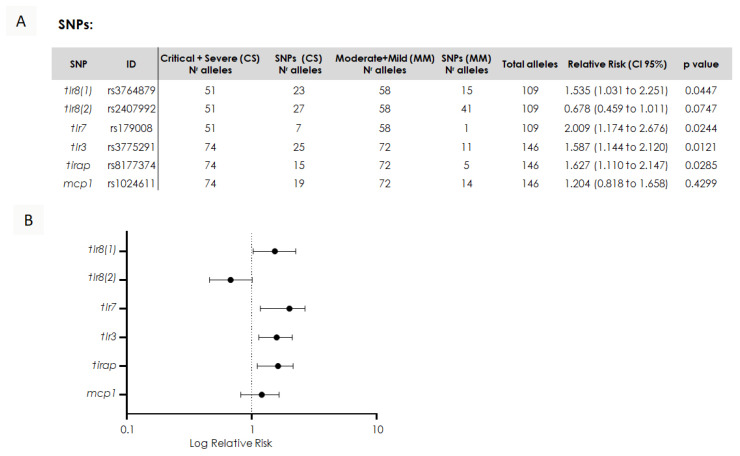
Association between SNPs and severity of symptoms in children with COVID-19. (**A**) Contingency that displays relative risk values with a confidence interval and *p* values between allele numbers in severe + critical symptom groups compared to allele numbers in the mild + moderate symptom group. (**B**) Forest plot showing the relative risk and 95% confidence interval for the presence of the mutation in the severe + critical symptom groups compared to the mild + moderate symptom group. Plots to the right of the dashed line indicate a positive association, and those to the left indicate a negative association; those crossing the dashed line indicate no association.

To investigate whether symptom progression was associated with SNPs, we conducted a relative risk analysis comparing the presence of mutations to the risk of more severe symptoms, as depicted in Figure 6. This analysis evaluated the probabilities of mutation presence between severe and critical symptom cases versus mild and moderate symptom cases. The analyzed data are presented in Figure 6A. The results indicated a higher relative risk of severe symptoms associated with SNPs in the *tlr8 (1)*, *tlr7*, *tlr3*, and *tirap* genes, with a *p* value smaller than 0.05. In contrast, no significant association was found for the SNP in the *tlr8 (2)* or *mcp1* gene, as shown in Figure 6A,B.

## 4. Discussion

We conducted a study on 73 children in Belo Horizonte (Brazil), all of whom were hospitalized at the state’s reference pediatric hospital and tested positive for COVID-19. The positivity of the infection was confirmed through various laboratory tests, including PCR, rapid tests, and antibody ELISA, along with the patient’s clinical presentation. While PCR is considered the gold standard for testing, we emphasize the importance of using rapid tests with clinical assessment during an epidemic. This approach allows clinicians to make quicker decisions.

The children who attended ranged from 0 to 13 years old, with a slight predominance of boys (*n* = 36, 50.7%). The highest concentration of ages was up to 2 years, with 49.3% (36 samples), and children under 1 year old accounted for 26.6%. Criteria such as ethnicity were disregarded due to the broad genetic diversity of the Brazilian population. A survey by Cimolai et al. in 2023 [39] reported that approximately 20% to 35% of hospitalized children are under 1 year old. The risk of needing intensive care also rises with younger age. However, the hospital admission rate might be higher for younger patients not necessarily due to more severe cases of COVID-19 but rather because of factors not directly related to the infection. These include the need for careful monitoring due to age-specific vulnerabilities and the precautions parents and doctors take. Additionally, comorbidities in children can significantly influence the course of COVID-19. When these comorbidities are combined with immune dysfunctions, they may further increase the risk of severe disease and hospitalization [40]. In our group study, we observed that 31% of the children had some comorbidity, including asthma, sickle cell anemia, heart disease, and trisomy 21, among others. Bain et al., 2023 [41], in a cohort study of 1303 children hospitalized in Brazil with COVID-19, reported that 10% had some comorbidity. Similarly, a study conducted in an outpatient clinic in Canada from August 2020 to July 2021 found that 5.5% of children had a pre-existing comorbidity [42].

The Eduardo de Menezes Hospital receives children for emergency care, addressing all levels of symptom complexity, and provides referrals for hospitalization or ICU if needed. We observed a median hospitalization time of 13 days, while the median intensive care time was 11. This duration is that reported by the CDC in 2023 [43], which indicated an average hospitalization time of 14 days for children in the U.S. According to Waghmare et al. (2023) [5], while most children are either asymptomatic or experience mild to moderate disease and recover within 1 to 2 weeks, complications such as respiratory distress syndrome, myocarditis, acute renal failure, and multisystem organ failure can still occur.

Our data encompass children with varying levels of severity, including those with severe outcomes and fatalities. We observed a high prevalence of diverse symptoms, such as respiratory syndrome, gastroenteritis, peritonitis, acute generalized lymphadenitis, acute renal failure, and otitis media. This diverse array of manifestations highlights the complexity of the disease. It emphasizes the need for further investigation into factors that may influence the severity and progression of COVID-19 in pediatric patients, including genetic variations. The allelic frequency of SNPs in the coding genes *tlr8 (1)*, *tlr8 (2)*, *tlr7, tlr3*, *tirap*, and *mcp1* was calculated for the population of hospitalized children with COVID-19 and compared with global databases such as Global ALFA, Global 1000 Genomes, and Global gnomAD, as well as databases with populations more closely related to South America, such as Americana 1000 Genomes and Americana gnomAD. Global ALFA, Global 1000 Genomes, and Global gnomAD are databases that provide unified genetic information from diverse populations worldwide. Americana 1000 Genomes and Americana gnomAD, although focused on the Americas, also aggregate data from distinct population groups. These populations are from countries of North, Central, and South America, as well as Indigenous populations (1000 Genomes Project Consortium: the gnomAD Consortium).

Since Brazil is a highly ethnically mixed country, which exhibits unique characteristics in the Americas, it necessitates a more critical approach when comparing its data with broader datasets. The comparison of SNP frequencies in hospitalized children with those in the databases showed that the genes *tlr7*, *tlr3*, and *mcp1* had lower frequencies, particularly *tlr7*, where the difference was statistically significant. At first glance, this result may seem contradictory. However, it is important to analyze the symptom severity alongside the frequency distribution of SNP in the studied population.

Our frequency analyses were categorized for comparative analysis by sex (Figure 4). Cumulative frequency data show that girls had more SNP alleles than boys (≥3 SNPs, 55.55% vs. 35.1%, respectively). The comparison of SNP frequencies by sex revealed the most significant differences for the SNPs tested in *tlr8 (1)* and *mcp1*. Subsequently, we showed that *tlr8 (1)* was associated with severe symptom cases. However, *mcp1* showed an inconsistent distribution, even when analyzed alongside other data.

In dividing children into categories, we observed that in the mild-to-moderate-symptom cases, there was a higher percentage of girls (61.1%). In contrast, in severe and critical symptom cases, the number of boys was predominant (62.2%). It is also worth noting that all four deaths were among boys. A study by Alwani et al. (2021) [44] indicated that males tend to experience more severe cases and higher mortality from COVID-19. Possible explanations for this difference include immunological, genetic, and hormonal factors. The X chromosome contains crucial immune function genes, such as *tlr8* and *tlr7*. In girls, X chromosome inactivation results in a mix of cells with either the maternal or paternal X chromosome inactivated, known as ‘cell mosaicism’. This mosaicism allows women to better adapt to infections and express functional alleles despite X-linked mutations.

Additionally, some immune-related genes are partially reactivated in female lymphocytes, enhancing their immune response to infections [45,46,47]. When comparing the frequency of SNPs across each severity symptom level (mild, moderate, severe, and critical), we observed that the SNPs in the *tlr8 (1)*, *tlr7*, *tlr3*, and *tirap* genes were more concentrated in the more severe symptom categories. *tlr8 (1)* and *tlr3* showed a gradual increase in frequency, while SNPs in *tlr7* and *tirap* were more concentrated in the severe symptom categories. These data suggest a potential association between severe symptom cases and SNPs in *tlr8 (1)*, *tlr7*, *tlr3*, and *tirap* genes.

When analyzing the deaths, we found that out of four cases, one had a mutation for *tlr7*, one for *tlr3*, and two for *tlr8 (2)*. However, while the overall frequency of the *tlr7* SNP in our study population was 7.52%, the *tlr8 (2)* SNP has a frequency of 65.94%. Thus, one out of four deaths (25%) in *tlr7* SNPS, compared to an overall frequency of 7.52%, suggests a probable association between severe symptoms and this SNP, whereas 50% of the deaths in SNPS *tlr8 (2)*, compared to an overall frequency of 65.9%, appear to be more of a random event. Furthermore, the distribution of the *tlr8 (2)* SNP across different symptom severity categories (Figure 5A) indicates that this SNP does not seem related explicitly to symptom severity. These data corroborate the relative risk analyses comparing mild + moderate with severe + critical symptom cases (severe + critical and deaths) (Figure 6).

The association of the rs3764879 [*tlr8 (1)*] mutation and increased susceptibility to infection have already been reported for some RNA viruses such as hepatitis C virus, Dengue virus, Chikungunya virus, DNA viruses such as Epstein Barr virus, and bacteria such as *Mycobacterium tuberculosis* [30,42,48,49,50,51].

A positive relationship has been observed between the presence of the T allele (rs179008) and an 89% increased risk of hepatitis C virus infection (for homozygotes) [30]. Some studies in the context of the COVID-19 pandemic have shown that *tlr7* (rs179008) polymorphisms (in *tlr7* gene) were significantly associated with an increased risk of pneumonia [42], increasing the risk of developing critical COVID-19 by 16 times for men with this mutation.

Our analysis reveals a statistically significant association between *tlr3* (rs3775291) mutation and infection susceptibility. Notably, Barkhash et al., 2013 [51] reported that the G allele of rs3775291 is linked to tick-borne encephalitis in the Russian population.

Regarding the *tirap* polymorphism rs8177374, our data showed a higher presence of this polymorphism in children with severe and critical symptoms. Research by Song et al. (2010) [52] has indicated that genetic variants in the *tirap* gene could influence susceptibility to sepsis-associated acute lung injury within the Han Chinese population.

Finally, mutations in gene *mcp1 rs1024611* have mainly been studied in relation to coronary artery disease, varicose veins [53,54], and atherosclerosis [53]. There are few studies focused on infectious diseases, particularly viral ones, highlighting the need for more research on this polymorphism. Our work did not identify a relationship between this mutation and severe and critical symptoms in children with COVID-19.

The limitation of this study is the small sample size of patients, as children were less affected by COVID-19 compared to adults. This may also be due to the mildness of infections in children and the lack of internal control in this study.

## 5. Conclusions

This study indicated a greater relative risk of severe symptoms in children with COVID-19, particularly associated with mutations in the SNPs *tlr8 (1)*, *tlr7*, *tlr3*, and *tirap*, with a significance level of *p* < 0.05. The prevalence of the *tlr7* mutation was lower compared to all five global groups with which we made comparisons. In our study, we identified one case of death among children infected with SARS-CoV-2 associated with the *tlr7* SNP, which accounted for 25% of the deaths. Similarly, there was another case of death among children infected with SARS-CoV-2 linked to the *tlr3* SNP, also representing 25% of the deaths in our study. These findings suggest that there is an increased relative risk of severe symptoms in children with COVID-19, associated with mutations in the *tlr7* and *tlr3* genes. Together, these two SNPs were associated with 50% of deaths.

## Figures and Tables

**Table 1:** Identification of mutation sites and most frequent nucleotides. *[1]*, *[2]*, and *[3]* primers were designed by the authors; *[4]*—rs3775291 [32]; *[5]*—rs8177374 [33]; *[6]*—rs1024611 [34].

*Gene Ref.*		*Primers*	*Tm*	*SNP*	*Location*	*Nucleotide Change*
*tlr8 (1) [1]*	F	GGCAACAGCTAAGAAATCAC	54 °C	rs3764879	chrX: 12906578	129 C>G
	R	CGCTTTACCTGCATTTACAGT				
*tlr8 (2) [2]*	F	GGTAGAATTAGTTTTCAGTGGCAA	54 °C	rs2407992	chrX: 12920993	2040 G>C
	R	GGTGGTGGTCTTAGTTTCAA				
*tlr7 [3]*	F	AGAGAGGCAGCAAATGGGAA	55 °C	rs179008	chrX: 12885540	11 A>T
	R	TAGGAAACCATCTAGCCCCA				
*tlr3 [4]*	F	TCTTGGGACTAAAGTGGACA	55 °C	rs3775291	chr4: 186082920	412 C>T
	R	CCCAACCAAGAGAAAGCATC				
*tirap [5]*	F	GGTGCAAGTACCAGATGCT	58 °C	rs8177374	chr11: 126292948	180 C>T
	R	CAACGCATGACAGCTTCTTT				
*mcp1 [6]*	F	CTTTCCCTTGTGTGTCCCC	58 °C	rs1024611	chr17: 34252769	−2518 A>B
	R	ACAGTAAACACAGGGAAGGT				

## Data Availability

Data are available at request to authors.
